# Supplementary material for: The Systems Biology Research Tool: evolvable open-source software
Source: BMC Syst Biol. 2008 Jun 29;2:55. doi: 10.1186/1752-0509-2-55 (PMC2446383; doi:10.1186/1752-0509-2-55)
Supplement: Additional file 1 — SBRT Archive. An archive of the current version of the Systems Biology Research Tool. [file 1752-0509-2-55-S1.zip › sbrt-1.4.0/doc/developers_guide/api/sbrt/shell/text/MapFormat.html]

MapFormat


|  |  |  |
| --- | --- | --- |
| **Overview** \| **Package** \| **Class** \| **Use** \| **Tree** \| **Deprecated** \| **Index** \| **Help** | |  |
| **PREV CLASS**   **NEXT CLASS** | **FRAMES**    **NO FRAMES**     **All Classes** |  |
| SUMMARY: NESTED \| FIELD \| CONSTR \| METHOD | DETAIL: FIELD \| CONSTR \| METHOD |  |


---


## sbrt.shell.text Interface MapFormat<K,V>

**Type Parameters:**: `K` - the key type.: `V` - the value type.

**All Superinterfaces:**: Format

**All Known Subinterfaces:**: AppInputFileLineFormat, ConstraintsFileLineFormat<E>, FbaOptFileLineFormat, FluxCapFormat, FluxomeSolutionFileLineFormat, FluxVectorFormat

**All Known Implementing Classes:**: AbstractMapFormat, AppInputFileLineFormatVersion1, ColonMapFormatV1, ConstraintsFileLineFormatV1, ConstraintsFileLineFormatV2, EqualsMapFormatV1, FbaOptFileLineFormatV1, FluxCapFormatV1, FluxomeSolFileLineFormatV1, FluxVectorFormatV1, IrfFormatV1

---

``` public interface MapFormat<K,V> extends Format ```

This interface is used to represent formats for maps.

There are multiple ways to represent maps as strings.
One way is to store the key-value pairs as a single
unit, such as *key = value*. Another way is to
store the keys separately from their values, such
as

*key_1_ ... key_n_*

*value_1_ ... value_n_*

This interface provides methods to deal with these
possibilities.

**Author:**
:   This interface was written and documented by
    Jeremiah Wright while in the Wagner lab.

---

| **Method Summary** | |
| --- | --- |
| `<T extends K> java.lang.String` | `formatKey(T key)`             Returns a formatted string representation of the provided key. |
| `java.lang.String` | `formatKeys(java.util.Set<? extends K> keys)`             Returns a formatted string representation of the provided set of keys. |
| `java.lang.String` | `formatPair(java.util.Map.Entry<? extends K,? extends V> keyValuePair)`             Returns a formatted string representation of the provided key-value pair. |
| `<S extends K,T extends V> java.lang.String` | `formatPair(S key, T value)`             Returns a formatted string representation of the provided key-value pair. |
| `<T extends V> java.lang.String` | `formatValue(T value)`             Returns a formatted string representation of the provided value. |
| `java.lang.String` | `formatValues(java.util.List<? extends V> values)`             Returns a string representation of the provided list of values. |
| `java.lang.String` | `formatValues(java.util.Map<? extends K,? extends V> map)`             Returns a string representation of the values in the provided map formatted as a list. |
| `java.util.LinkedHashSet<K>` | `parseAllKeys(java.lang.String allKeys)`             Parses the provided string and returns its corresponding set of keys. |
| `java.util.Map<K,V>` | `parseAllPairs(java.lang.String allKeys, java.lang.String allValues)`             Parses the provided set of keys and list of values and returns a corresponding map of key-value pairs. |
| `java.util.Map<K,V>` | `parseAllValues(java.util.LinkedHashSet<? extends K> keys, java.lang.String allValues)`             Parses the provided string of values and returns a corresponding map of key-value pairs using the provided set of keys. |
| `java.util.List<V>` | `parseAllValues(java.lang.String allValues)`             Parses the provided string and returns its corresponding list of values. |
| `K` | `parseKey(java.lang.String key)`             Parses the provided string and returns its corresponding key. |
| `java.util.Map.Entry<K,V>` | `parsePair(java.lang.String keyValuePair)`             Parses the provided string and returns a corresponding map entry. |
| `V` | `parseValue(java.lang.String value)`             Parses the provided string and returns its corresponding value. |

**Method Detail**

### formatKey

```
<T extends K> java.lang.String formatKey(T key)
```

:   Returns a formatted string representation of the
    provided key.

    :   **Type Parameters:**: `T` - a type that extends the primary key type of this format. **Parameters:**: `key` - the key to be formatted. **Returns:**: a formatted string representation of the provided key.

---


### formatValue

```
<T extends V> java.lang.String formatValue(T value)
```

:   Returns a formatted string representation of the
    provided value.

    :   **Type Parameters:**: `T` - a type that extends the primary value type of this format. **Parameters:**: `value` - the value to be formatted. **Returns:**: a formatted string representation of the provided value.

---


### formatKeys

```
java.lang.String formatKeys(java.util.Set<? extends K> keys)
```

:   Returns a formatted string representation of the
    provided set of keys.

    :   **Parameters:**: `keys` - the set of keys to be formatted. **Returns:**: a formatted string representation of the provided set of keys.

---


### formatValues

```
java.lang.String formatValues(java.util.List<? extends V> values)
```

:   Returns a string representation of the
    provided list of values.

    :   **Parameters:**: `values` - the list of values to be formatted. **Returns:**: a formatted string representation of the provided list of values.

---


### formatValues

```
java.lang.String formatValues(java.util.Map<? extends K,? extends V> map)
```

:   Returns a string representation of the
    values in the provided map formatted as a list. The order of
    occurrence of values in the returned string depends
    on the *order* of the map. See
    `java.util.Map` for further details.

    :   **Parameters:**: `map` - keys mapped to their respective values. **Returns:**: a string representation of the values in the provided map formatted as a list.

---


### formatPair

```
<S extends K,T extends V> java.lang.String formatPair(S key,
                                                      T value)
```

:   Returns a formatted string representation of the
    provided key-value pair.

    :   **Type Parameters:**: `S` - a type extending the primary key type.: `T` - a type extending the primary value type. **Parameters:**: `key` - the key.: `value` - the value. **Returns:**: a formatted string representation of the provided key-value pair.

---


### formatPair

```
java.lang.String formatPair(java.util.Map.Entry<? extends K,? extends V> keyValuePair)
```

:   Returns a formatted string representation of the
    provided key-value pair.

    :   **Parameters:**: `keyValuePair` - the key-value pair to be formatted. **Returns:**: a formatted string representation of the provided key-value pair.

---


### parseKey

```
K parseKey(java.lang.String key)
```

:   Parses the provided string and returns its
    corresponding key.

    :   **Parameters:**: `key` - a string representation of a key. **Returns:**: the key corresponding to the provided string.

---


### parseValue

```
V parseValue(java.lang.String value)
```

:   Parses the provided string and returns its
    corresponding value.

    :   **Parameters:**: `value` - a string representation of a value. **Returns:**: the value corresponding to the provided string.

---


### parsePair

```
java.util.Map.Entry<K,V> parsePair(java.lang.String keyValuePair)
```

:   Parses the provided string and returns a
    corresponding map entry.

    :   **Parameters:**: `keyValuePair` - a string representation of a key-value pair, or in other words, a map entry. **Returns:**: a map entry containing the key-value pair corresponding to the provided string.

---


### parseAllKeys

```
java.util.LinkedHashSet<K> parseAllKeys(java.lang.String allKeys)
```

:   Parses the provided string and returns its
    corresponding set of keys.

    :   **Parameters:**: `allKeys` - a string representation of a set of keys. **Returns:**: a set of keys corresponding to the provided string.

---


### parseAllValues

```
java.util.List<V> parseAllValues(java.lang.String allValues)
```

:   Parses the provided string and returns its
    corresponding list of values.

    :   **Parameters:**: `allValues` - a string representation of a list of values. **Returns:**: a list of values corresponding to the provided string.

---


### parseAllValues

```
java.util.Map<K,V> parseAllValues(java.util.LinkedHashSet<? extends K> keys,
                                  java.lang.String allValues)
```

:   Parses the provided string of values and returns a
    corresponding map of key-value pairs using the
    provided set of keys. The association between
    keys and values is determined from their order of
    occurrence in their respective collection. In other
    words, the first key returned by the set's iterator
    is associated with the first parsed value, and so on.

    :   **Parameters:**: `keys` - the set of keys with which to associate the parsed values.: `allValues` - a string representation of a list of values. **Returns:**: keys mapped to their respective values.

---


### parseAllPairs

```
java.util.Map<K,V> parseAllPairs(java.lang.String allKeys,
                                 java.lang.String allValues)
```

:   Parses the provided set of keys and list of values
    and returns a corresponding map of key-value pairs.
    The association between keys and values is determined
    from their order of occurrence in their respective collection.
    In other words, the first parsed key is
    associated with the first parsed value, and so on.

    :   **Parameters:**: `allKeys` - a string representation of a set of variables.: `allValues` - a string representation of a list of values. **Returns:**: keys mapped to their respective values.


---


|  |  |  |
| --- | --- | --- |
| **Overview** \| **Package** \| **Class** \| **Use** \| **Tree** \| **Deprecated** \| **Index** \| **Help** | |  |
| **PREV CLASS**   **NEXT CLASS** | **FRAMES**    **NO FRAMES**     **All Classes** |  |
| SUMMARY: NESTED \| FIELD \| CONSTR \| METHOD | DETAIL: FIELD \| CONSTR \| METHOD |  |


---
